# Pharmacokinetics of mycophenolic acid and its effect on CD4^+^ and CD8^+^ T cells after oral administration of mycophenolate mofetil to healthy cats

**DOI:** 10.1111/jvim.15585

**Published:** 2019-08-19

**Authors:** Jennifer E. Slovak, Julianne K. Hwang, Sol M. Rivera, Nicolas F. Villarino

**Affiliations:** ^1^ Department of Veterinary Clinical Sciences, College of Veterinary Medicine Washington State University Pullman Washington

**Keywords:** cats, immunosuppressant, mycophenolate mofetil, pharmacodynamics, pharmacokinetics

## Abstract

**Background:**

Mycophenolate mofetil (MMF) is an immunosuppressant used in human and veterinary medicine. Little pharmacokinetic and pharmacodynamic information on MMF is available in cats.

**Objective:**

To evaluate the plasma disposition of mycophenolic acid (MPA) and assess its effect on total peripheral blood mononuclear cells and CD4^+^/CD8^+^ ratios after PO administration of MMF.

**Animals:**

Healthy cats (n = 10).

**Methods:**

Mycophenolate mofetil was administered at a dosage of 10 mg/kg q12h (n = 3), 15 mg/kg q12h (n = 3), and 15 mg/kg q8h (n = 4) for 7 days. Concentrations of MPA and derivatives were determined using ultra‐high‐performance liquid chromatography. Flow cytometry was used to assess CD4^+^/CD8^+^ T‐cell ratios.

**Results:**

All cats biotransformed MMF into MPA. Half of the cats (5/10) had adverse effects within 1 week of MMF administration. Area under the curve limit of quantification (AUC_0‐LOQh_) of MPA ranged from 1.27 to 2.03 hours·μg/mL and from 1.77 to 8.54 hours·μg/mL after the first and last PO dose of 10 mg/kg. The AUC_0‐loqh_ of MPA ranged from 2.18 to 31 hours·μg/mL after the first dose of 15 mg/kg of MMF. Before the first dose of MMF, the average total number of PBMC ranged from 1.2 to 9.3 million/mL. At the last dose of MMF, the average total number of PBMC ranged from 3 to 5 million/mL.

**Conclusion:**

Mycophenolic acid was detected in all cats. The dose 10 mg/kg given q12h for 1 week was tolerated (n = 3). The efficacy of MMF as an immunosuppressant and long‐term safety in cats of this dosage regimen is unknown.

**Abbreviations**
AUC_0‐loqh_: observed area under the drug concentration vs time curve from zero to the last quantifiable concentration within the first or last dose interval
IMPDH: inosine monophosphate dehydrogenase
LLOQ: lower limit of quantification
MMF: mycophenolate mofetil
MPA: mycophenolic acid
MPAG: MPA phenol glucuronide
MPAGls: MPA phenol glucoside
PBMC: peripheral blood mononuclear cell counts
PBS: phosphate‐buffered saline
RPMI: Roswell Park Memorial Institute Media
SNP: single nucleotide polymorphism

## 1 INTRODUCTION

Few orally administered immune suppressants are available in clinical veterinary medicine despite many conditions requiring their use. Many immune‐mediated diseases that occur in veterinary medicine are unpredictable and impact the patient's quality of life.^1^, ^2^, ^3^ Few investigations into the use of alternative medications in cats have been performed. Therefore, further research is warranted for clinically relevant and easily administered PO immunosuppressant drug options for cats.

Mycophenolate mofetil is an immunosuppressant used in human medicine in organ transplantation patients.^4^, ^5^, ^6^, ^7^ It has been used in veterinary medicine^8^, ^9^, ^10^, ^11^, ^12^ as a secondary immunosuppressant with little published research in cats.^11^, ^13^, ^14^ To date, safe and effective dosage regimens remain to be established. Mycophenolate mofetil is a prodrug of the active moiety mycophenolic acid (MPA), a fermentation product of *Penicillium*.^7^, ^15^ After PO administration, MMF undergoes rapid presystemic tissue de‐esterification^15^, ^16^, ^17^ and is converted to the active metabolite MPA.^15^ In cats, MPA is highly bound to plasma proteins,^18^ and is eliminated from the body rapidly likely by hepatic biotransformation into at least 2 metabolites: MPA phenol glucoside (MPAGls) and MPA phenol glucuronide (MPAG).^19^, ^20^

The primary action of MPA involves decreasing T and B lymphocyte proliferation by a specific and noncompetitive mechanism of action that decreases production of antibodies, decreases proliferation of CD4^+^ and CD8^+^ lymphocytes, and inhibits adhesion of glycoproteins to endothelial cells. 15, 21 These effects occur by inhibiting inosine monophosphate dehydrogenase (IMPDH), the rate‐limiting enzyme in de novo guanosine synthesis.^15^, ^21^ The disposition of MPA in cats has been studied after IV infusion of MMF,^19^ suggesting that the disposition of MPA is highly variable. In addition, MMF administered IV at a dosage of 10 mg/kg q12h for 3 days in healthy cats resulted in little change in total peripheral blood mononuclear cell (PBMC) counts after MMF administration.^20^ Despite preliminary data regarding IV administration of MMF, it remains to be determined if MMF is absorbed after PO administration to cats. The disposition of MPA after repeated PO administration, its effect on PBMCs, and immune effector cells such as CD4^+^ and CD8^+^ lymphocytes and their relevance to MMF administration have yet to be described. Our purpose was to characterize the disposition of MPA after PO administration of MMF at 10 mg/kg q12h, 15 mg/kg q12h, and 15 mg/kg q8h for up to 7 days to healthy cats and to evaluate the effect of these dosage regimens on total PBMC and CD4^+^ and CD8^+^ lymphocytes 24, 48, 168, and 180 hours after the initial PO dose.

## 2 MATERIALS AND METHODS

### 2.1 Animal population

The research protocol was approved by the Washington State University Institutional Animal Care and Use Committee (ASAF # 04665‐005). Ten healthy adult (1‐5 years old) cats (5 spayed females and 5 neutered males), weighing 4‐7.4 kg (median, 4.9 kg) were included. Eight cats were domestic shorthairs and 2 were domestic longhair cats. The cats were housed according to Washington State University Institutional Animal Care and Use Committee guidelines. All cats were housed as individuals or in pairs in temperature‐ and humidity‐controlled enriched rooms. The cats were adopted at completion of the study.

All enrolled cats were deemed healthy and eligible based on prestudy physical examinations, behavior assessment (friendly, socialized) and a CBC, serum biochemistry, urinalysis, and feline leukemia virus/feline immunodeficiency virus screening (all tested negative). The study cats were acclimatized to their new environment and food (Purina Cat Chow indoor formula, Purina Animal Nutrition, Gray Summit, Missouri) for 7‐10 days before starting the study. Only 2 cats were medicated and sampled during the study at any one time. Twenty‐four hours before medication administration, the cats were sedated by IM injection of ketamine (Ketaset injectable [100 mg/mL], Zoetis Inc, Kalamazoo, Michigan) 5‐8 mg/kg, acepromazine (Promace injectable [10 mg/mL], Boehringer Ingelheim Vetmedica Inc, St Joseph, Missouri) 0.01‐0.03 mg/kg, and butorphanol (Torbugesic injectable [100 mg/mL], Boehringer Ingelheim Vetmedica Inc) 0.2‐0.4 mg/kg, and had an indwelling dual port jugular catheter placed by a licensed veterinary technician for blood sampling. The jugular catheters were irrigated with 1 mL of heparinized saline, q6‐8h.

### 2.2 Oral administration

Mycophenolate mofetil (Cellcept oral suspension MMF hydrochloride for injection [200 mg/mL], Genetech USA Inc, South San Francisco, California) was administered at a dosage of 10 mg/kg PO q12h for 7 days (n = 3), 15 mg/kg q12h for 7 days (n = 3), and 15 mg/kg PO q8h for 7 days (n = 4). The dosages were selected based on clinical experience and use of MMF, and information available in the literature for humans,^21^, ^22^ dogs,^8^, ^9^, ^12^ and cats.^19^, ^20^, ^23^ Food was withheld 2 hours before and after drug administration. Water was available ad libitum to the cats. A repeat CBC and serum biochemistry profile were performed in all study cats within 24 hours of the last PO MMF administration.

### 2.3 Blood collection

Blood was collected before administration of MMF for pharmacokinetic (PK) data at 0.25, 0.5, 1, 1.5, 3, 6, 8, 10, and 12 hours after the first PO dose. Additionally, blood samples were taken before the next dose at 24, 48, 72, 96, 120, and 144 hours after the initial PO dose. More intensive sampling also was done 156, 156.25, 156.5, 157, 157.5, 159, 162, 164, 166, and 168 hours after the initial dose. Blood samples for PK analysis were obtained and transferred to glass tubes containing citrate. The tubes were centrifuged at 1800*g* for 8 minutes. Plasma was separated and divided into 200 μL aliquots in Eppendorf (Eppendorf AG, Hamburg, Germany) tubes and stored at −80°C until analysis. Samples were analyzed in a single batch. For PBMC isolation, 1.5 mL of blood was collected and placed into glass tubes with lithium heparin before dosing, and 24, 144, and 168 hours after the initial MMF PO dose. A volume <5% of the circulating blood volume of the cats was obtained for analysis during the course of the study.

### 2.4 Determination of MPA, MPAG, and MPAGls

Plasma MPA and its derivatives MPAG (Sigma‐Aldrich Fine Chemicals, St. Louis, Missouri) and MPAGls (Sigma‐Aldrich Fine Chemicals) were quantified using an ultra‐high‐performance liquid chromatographic‐ultraviolet method validated in our laboratory.^24^, ^25^ The method was validated according to the Guidelines for Bioanalytical Method Validation published by the Food and Drug Administration (US Department of Health and Human Service, Food and Drug Administration; Center for Drug Evaluation and Research and Center for Veterinary Medicine Guidance for Industry; Bioanalytical Method Validation, May 2001; www.fda.gov/downloads/Drugs/Guidance).

Quality control samples (3 different concentrations) and calibration standards were prepared in feline plasma and run with the study samples. The calibration curve for the determination of MPA ranged between 0.3 and 20 μg/mL. The calibration curves for the determination of MPAG and MPAGls ranged between 0.3 and 3 μg/mL and 0.5 and 3 μg/mL, respectively. The calibration curves were linear (*R* ^2^>0.99), and the method was precise (coefficient of variation [CV] ≤15), and accurate (error [E] ≤15%. The lower limits of quantification (LLOQ) were 0.3, 0.25 and 0.2 μg/mL for MPA, MPAG, and MPAGls, respectively.

### 2.5 Pharmacokinetic analysis

Pharmacokinetic parameters were determined using non‐compartmental analyses. The arithmetic mean of PK parameters was obtained by averaging the individual parameter estimates. Maximum plasma concentration (C max) and time of maximum plasma concentration of MPA correspond to the observed maximal concentration and time of observed maximum concentration of MPA. Concentration at the end of the dosing interval corresponds to the concentration of MPA 12 hours after the administration of MMF. The area under the plasma concentration vs. time curve from 0 to LOQ (AUC_0‐loq_) corresponds to the area under the concentration vs. time curve from 0 hours to the latest time point within a dosing interval at which MPA was quantifiable (0.3 μg/mL). The AUC_0‐loq_ was estimated by use of the linear trapezoidal rule (sum of trapezoids) using the following equation as implemented by Phoenix WinNonlin v. 7 (Phoenix, version 7.1, Pharsight Corp Mountain View, California) where *t* is sampling time and *Y* is the observed outcome:

$$\mathrm{AUC}t1-t\mathrm{loq}=0.5\sum \left(\mathrm{Yi}+\mathrm{Yi}+1\right)\times \left(\mathrm{ti}+1-\mathrm{ti}\right).$$

The PK parameters were reported as mean and range unless otherwise noted. The AUC_0‐loq_ of MPA after the first and last administration of 10 mg/kg of MMF was compared statistically using a Mann‐Whitney test in GraphPad Prism v. 8. Significance level was *P* < .05.

### 2.6 Determination of the effect of the repeated administration of MMF

#### 2.6.1 PBMC preparation and analysis

The PBMCs were isolated from blood samples that were collected from the indwelling jugular catheter into tubes containing lithium‐heparin as an anticoagulant. Blood samples were allowed to stand undisturbed for 20 minutes at room temperature (21°C). Each blood sample was then diluted with an equal volume of phosphate‐buffered saline (PBS) solution. Diluted blood samples were carefully layered over commercially available gradient solution in 15 mL conical tubes at room temperature. Samples were centrifuged at 700*g* for 30 minutes at room temperature without braking during deceleration. The top layer was discarded, and the PBMC layer was collected from the gradient solution interface. The collected sample was washed, red blood cells were removed, and PBMCs were collected as described previously.^26^ The number of cells was counted by use of an automated thin‐film sensor cell counter (Orflo Moxi Z, Orlfo Technologies, Ketchum, Idaho) with cell count cassettes (Type S, Orlfo Technologies). The healthy cell population and viability were calculated by use of the internal curve‐fitting algorithm of the automated cell counter software.

#### 2.6.2 PBMC cryopreservation and thawing

The PBMCs were suspended at a concentration of 1 × 10^7^ cells/mL in 0.5 mL PBS solution at room temperature. Freezing medium 50% Roswell Park Memorial Institute Media (RPMI) 1640, 40% fetal bovine serum, and 10% dimethyl sulfoxide, at room temperature was added, and the suspension gently mixed. The resulting cell suspension was divided into two 1‐mL aliquots, which were placed in cryogenic polypropylene vials. The vials were placed into freezing containers (Mr. Frosty freezing containers, Thermo Fisher, Rochester, New York) containing an isopropyl alcohol medium; containers were placed into a freezer (−80°C) to achieve temperature lowering of approximately 1°C/min. Twenty‐four hours later, samples were transferred quickly into a liquid nitrogen (−196°C) and stored until testing.

Thawing of samples was accomplished by placing cryo‐vials in a 37°C water bath. As soon as samples were thawed, cells were pipetted into a 15‐mL conical tube containing 10‐fold amounts of warm complete RPMI medium (96.4% RPMI 1640, 2.5% heat‐inactivated fetal bovine serum, 1% penicillin‐streptomycin‐glutamine, and 0.1% 2‐mercaptoethanol). Cells were washed by centrifugation at 300*g* for 10 minutes at room temperature.

#### 2.6.3 Cell staining and flow cytometry

Cells were washed with complete RPMI medium and diluted to a concentration of 1 × 10^6^ cells/100 μL. Cells in the 2 aliquots (100 μL/aliquot) were pelleted by centrifugation at 1800*g* for 8 minutes and incubated with 100 μL of PBS solution and optimal concentrations of fluorescein isothiocyanate‐conjugated CD4^+^ (Mouse anti‐cat CD4:FITC, Bio‐Rad, Hercules, California) and phycoerythrin‐conjugated CD8^+^ (Mouse anti‐cat CD8 alpha/beta:RPE, Bio‐Rad) for 15 minutes at 4°C in the dark. Concentration of the antibodies was 1 μg of IgG/10 μL of PBS solution. Cells were washed twice with 200 μL of flow cytometry buffer (97% PBS solution and 3% heat‐inactivated fetal bovine serum) followed by centrifugation at 1800*g* for 4 minutes. Cells were resuspended in flow cytometry buffer, and lymphocytes were gated for characteristic forward‐ and side‐scatter profiles. We collected 25 000 total events per sample for flow analysis. The percentage of cells stained with antibody against CD4^+^ and CD8^+^ was determined as 2‐color flow cytometry profiles (BD FACSCalibur, BD Bioscience, San Jose, California). Percentages of stained cells were calculated by the use of flow cytometer software (FCS Express 4, BD Bioscience).

#### 2.6.4 Statistical analysis

Estimated PK parameters and T‐cell and total PBMC response to treatments were evaluated using descriptive statistics (GraphPad Prism, version 7, GraphPad Software Inc, San Diego, California).

## 3 RESULTS

All cats (4/4) had gastrointestinal signs (self‐limiting diarrhea and hyporexia) in the 15 mg/kg q8h group; no cats completed the trial. One cat in the 15 mg/kg q12h group had self‐limiting diarrhea; 2 of 3 cats completed the trial. No cats in the 10 mg/kg q12h group had diarrhea or hyporexia; 3 of 3 cats completed the trial. Once adverse effects were seen in affected cats, MMF was discontinued immediately. Serum biochemical results such as alanine transferase activity remained similar pre‐ and post‐MMF treatment. Platelet counts and PCV decreased in 9 of 10 cats post‐MMF treatment but remained adequate, based on blood smear slide evaluation by a clinical pathologist, as previously reported.^13^ No housing or food intake was altered in any of the cats during the study period.

### 3.1 Pharmacokinetic results

**Figure 1:**
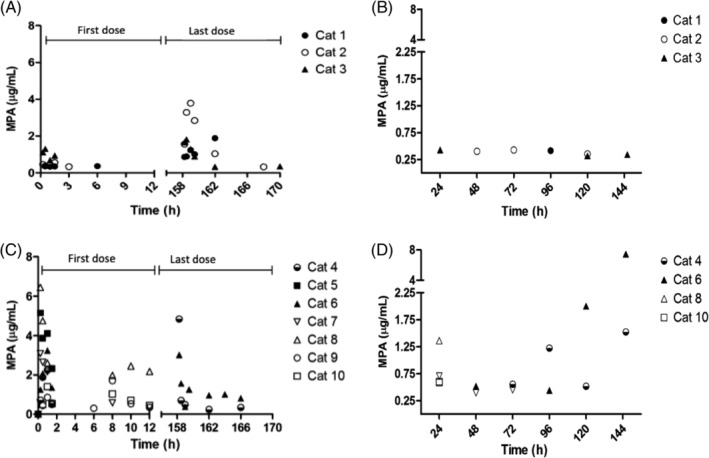
Disposition of MPA in plasma from cats. A, Following the administration of MMF at 10 mg/ kg after the first and last dose (n = 3). B, Trough values preceding administration of MMF every 12 hours at 10 mg/kg (n = 3), C, After administration of MMF at 15 mg/ kg q12h (n = 3) or q8h (n = 4) after the first and last dose. D, Trough values preceding the administration of MMF at 15 mg/ kg q12h (n = 3) or q8h (n = 4). Those cats not displayed in the figures correspond to cats with MPA plasma concentrations below the lower limit of quantification or did not complete the study

The disposition of MPA was evaluated after PO administration of MMF. After PO administration of MMF at 10 and 15 mg/kg, all cats biotransformed MMF to MPA (Table 1 and Figure 1). Pharmacokinetic parameters are presented in Table 1.

**Table 1:** PK parameters of MPA in plasma from cats following oral administration of MMF at 10 mg/kg every 12 hours and 15 mg/kg every 12 hours and every 8 hours for up to 7 days

Dose		Individual				
10 mg/kg	Pharmacokinetic parameter	Cat 1	Cat 2		Cat 3	Mean
After first dose (n = 3)	Time to maximum concentration (h)	0.5	1.5		0.5	0.5
	Observed maximum concentration (μg/mL)	0.35	0.55		1.3	0.73
	Concentration at 12‐h post‐MMF administration (μg/mL)	BLLOQ	BLLOQ		BLLOQ	NA
	AUC_0–loq_ (h·μg/mL)	2.03	1.28		1.35	1.55
After last dose (n = 3)	Time to maximum (h)	0.5	0.5		0.5	0.5
	Observed maximum concentration (μg/mL)	1.23	3.78		1.88	2.29
	Concentration at 12‐h post MMF administration (μg /mL)	BLLOQ	BLLOQ		0.34	NA
	AUC_0–loq_ (h·μg /mL)	1.77	8.54		4.56	4.95
**15 mg/kg q12h**	**Pharmacokinetic parameter**	**Cat 4**	**Cat 5**		**Cat 6**	**Mean**
After first dose (n = 3)	Time to maximum (h)	1	1		1	1
	Observed maximum concentration (μg/mL)	2.24	4.11		3.24	3.19
	Concentration at 12‐h post‐MMF administration (μg /mL)	BLLOQ	BLLOQ		0.51	NA
	AUC_0–loq_ (h·μg/mL)	2.15	5.37		5.47	4.33
After last dose (n = 2)	Time to maximum (h)	0.25	NA		0.25	0.25
	Observed maximum concentration (μg/mL)	4.84	NA		3.01	3.92
	Concentration at 12‐h post MMF administration (μg /mL)	BLLOQ	NA		BLLOQ	NA
	AUC_0–loq_ (h·μg /mL)	2.52	NA		6.03	4.27
**15 mg/kg q8h**	**Pharmacokinetic parameter**	**Cat 7**	**Cat 8**	**Cat 9**	**Cat 10**	**Mean**
After first dose (n = 4)	Time to maximum (h)	0.25	0.5	1.5	1.5	0.93
	Observed maximum concentration (μg/mL)	3.0	6.63	0.86	1.40	2.97
	Concentration at 12‐h post MMF administration (μg /mL)	0.59	1.36	0.70	0.60	0.81
	AUC_0–loq_ (h·μg /mL)	14.9	31.0	5.57	9.48	15.2

Abbreviations: BLLOQ, below lower limit of quantification (0.3 μg/mL); NA, not applicable.

For 10 mg/kg dosage regimen protocol, the median observed maximum plasma concentration and AUC_0‐loq_ after the first and last dose were not different (*P* = .2).

**Figure 2:**
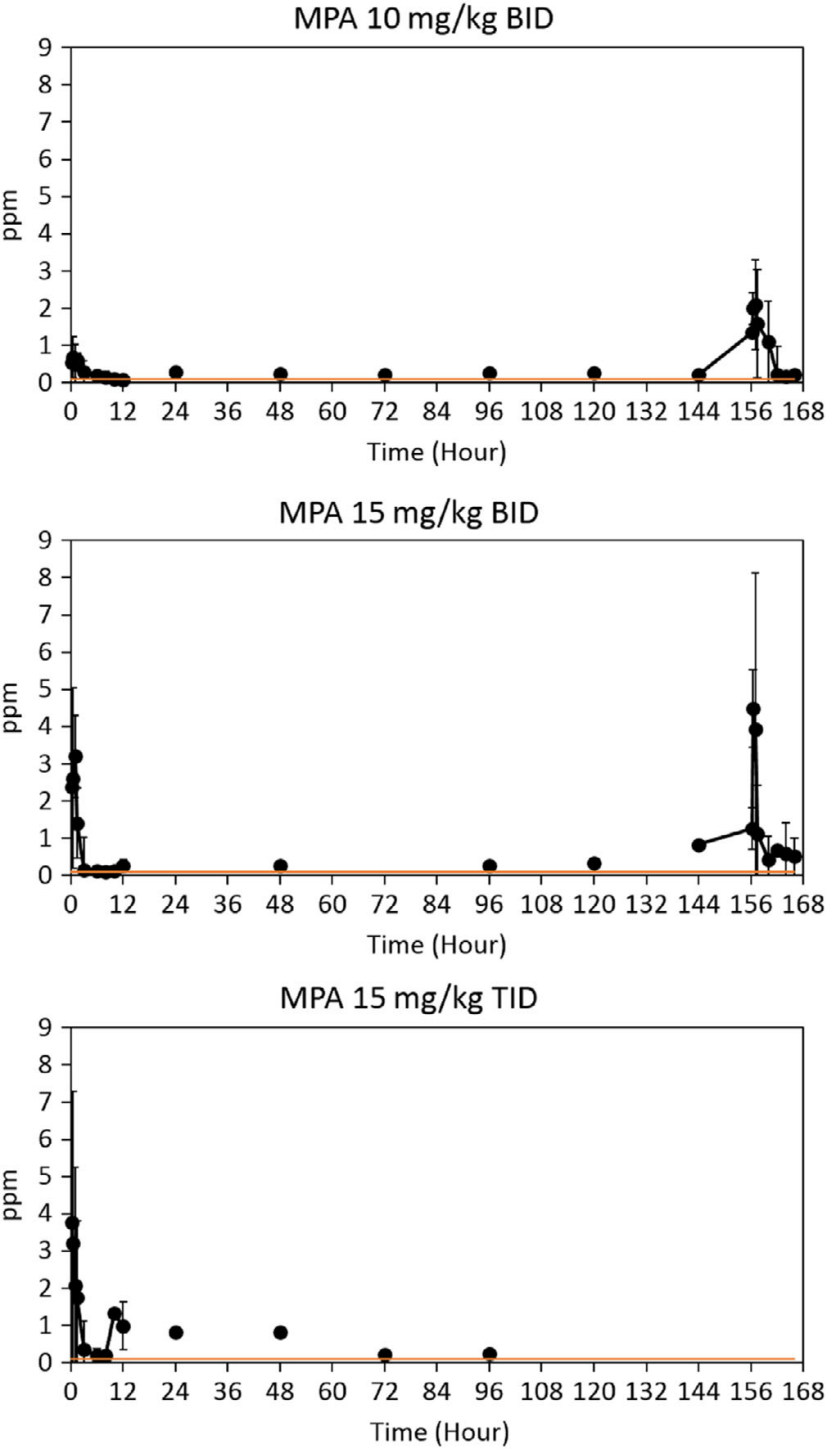
MPA concentrations for all treatment groups. *Orange line reflects that many values shown were below the lower limit of quantification but above the lower limit of detection

**Figure 3:**
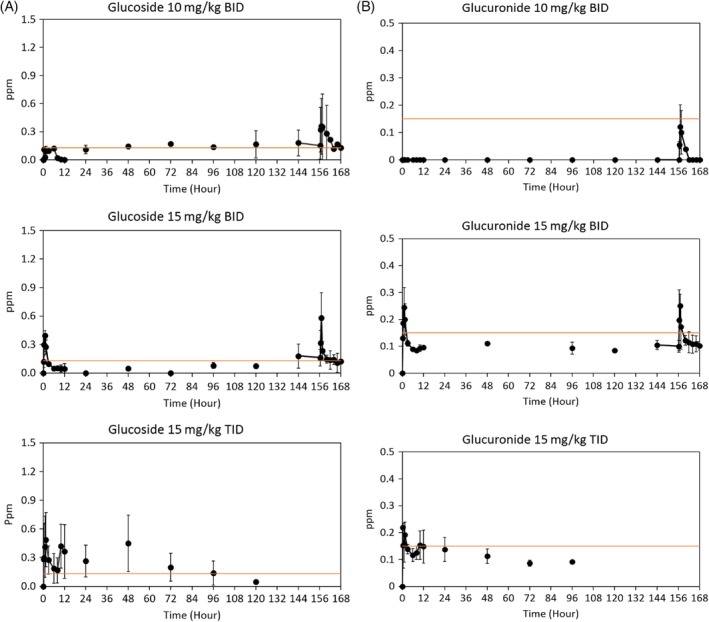
A, MPAGls concentrations for all treatment groups. *Orange line reflects that many values shown were below the lower limit of quantification but above the lower limit of detection. B, MPAG concentrations for all treatment groups. *Many values shown were below the lower limit of quantification but above the lower limit of detection

For all treatment groups, MPAG and MPAGls were detected in all cats, but the concentrations were below the validated LLOQ in some cats. After the last administration of 10 mg/kg q12h MMF, 1 of 3 cats had quantifiable concentrations of MPAGls ranging from 0.61 to 0.75 μg/mL (all cats finished the study). Only 1 cat treated with 15 mg/kg MMF q12h had quantifiable concentrations of MPAGls (range, 0.61‐0.75 μg/mL) after the last MMF dose (2 cats finished the study). Two cats included in the 15 mg/kg of MMF q8h group had quantifiable concentrations of MPAGls (range, 0.54‐0.85) after the first or second dose of MMF; none of the cats completed the study (Figures 2 and 3).

### 3.2 Determination of the effect of the repeated administration of MMF

Cell counting was assessed by use of the trypan blue dye exclusion test.^27^ We assessed cell viability using the Moxi population index; all tested samples had an average of 95%‐98% viability.

**Figure 4:**
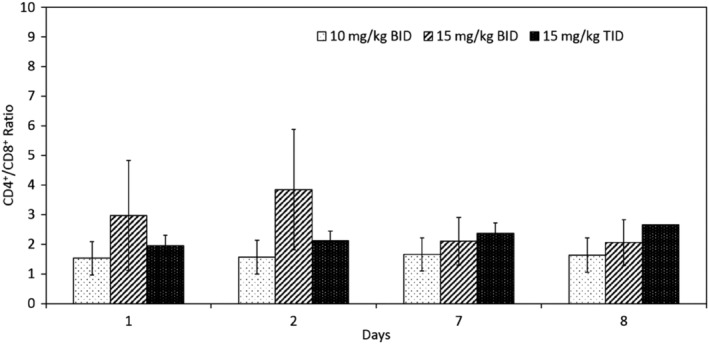
CD4^+^/CD8^+^ ratios (mean ± standard deviation) for all cats (n = 10) given different doses of MMF on days 1, 2, 7, 8

**Figure 5:**
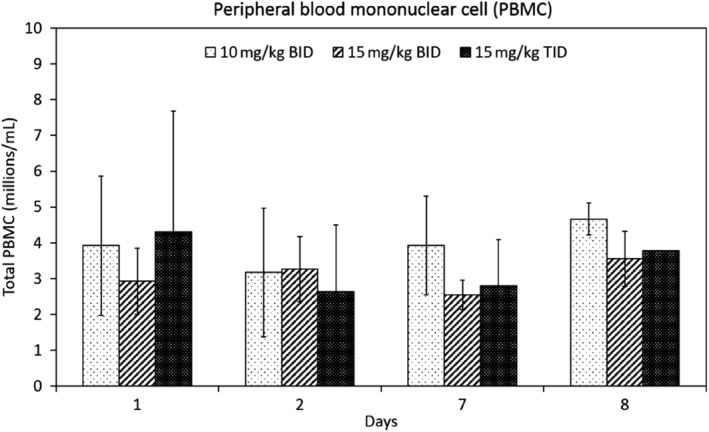
Total PBMC (mean ± standard deviation) for all cats (n = 10) given different doses of MMF on days 1, 2, 7, 8

Total isolated PBMC counts were variable in all cats before MMF administration and 24, 144, and 168 hours after PO MMF dose (Figure 4). The total number of PBMC was relatively static at all time points for all treatment groups (Figure 4). The average total number of PBMC pre‐dose ranged from 1.2‐9.3 × 10^6^/mL and end‐dose ranged from 3‐5 × 10^6^/mL. No appreciable change occurred in CD4^+^ or CD8^+^ cells in any treatment group (Figure 5). Only 1 of 10 cats had a noticeable change in the CD4^+^/CD8^+^ ratio from 5.56 at pre‐dose to 2.6 at 168 hours after the initial MMF (12 hours after the last PO dose of MMF). All other cat lymphocyte ratios either remained static or increased (Figure 5). Statistical evaluation was not performed on this data because of the small number of cats in each group.

## 4 DISCUSSION

We report the disposition of MPA in plasma after PO administration of 10 mg/ kg q12h, 15 mg/kg q12h, and 15 mg/kg q8h of MMF for up to 1 week in healthy cats. Additionally, our study provides information about the short‐term in vivo effects of PO administration of MMF on total PBMC counts and CD4^+^ and CD8^+^ lymphocytes at several time points before, during, and after MMF administration.

All cats treated with 15 mg/g q8h had adverse gastrointestinal effects including self‐limiting diarrhea and normal feces within 24 hours of stopping MMF. Similar observations have been reported in in humans and cats.^19^, ^20^, ^21^

Mycophenolic acid was detected in plasma of all the cats within 2 hours after PO administration of MMF, indicating that the prodrug MMF was absorbed and biotransformed to MPA. The metabolites MPAGls and MPAG were detected in plasma of all cats, suggesting that MPA was eliminated, at least partially, by hepatic biotransformation. These observations are consistent with those of previous studies.^19^, ^20^, ^23^

The plasma disposition of MPA was variable in the cats as was the peak concentration of MPA (Figure 1 and Table 1). This variability may be the result of variable absorption of MMF and subsequent metabolism in each cat. The disposition of MPA in plasma resulting in high inter‐cat variability is not a phenomenon exclusive to cats and also has been reported in humans.^21^ It remains to be determined if the interindividual variability of MPA observed in plasma has any impact on MPA's pharmacological effect. The trough MPA plasma concentrations (Figure 1B, D) suggest that MPA accumulates in plasma in some cats after administration of MMF at a dosage of 15 mg/kg. In humans, optimal plasma concentrations of MPA suggest a target of MPA AUC_12h_ > 35 mg·h/L, or AUC_0‐12h_ of 30‐60 mg·h/L leading to better efficacy and outcomes.^21^ In our study, mean AUC_0‐loqh_ after the first and last dose of MMF at 10 and 15 mg/kg was relatively smaller (Table 1) than the AUC_0‐12h_ proposed as optimal for inducing an immunomodulatory effect in humans, suggesting that our chosen dosage regimens for MMF might not result in effective MPA exposure.

Decreasing lymphocyte proliferation is a crucial effect of MMF administration and is concentration‐dependent.^5^, ^28^ Regulating an inappropriate immune response by inhibition of T cells is important because T cells contribute to autoimmune diseases by multiple stimulatory, activation, and cytotoxic mechanisms.^29^, ^30^ In our study, total PBMC numbers of the cats remained largely unaffected by the PO MMF, as did CD4^+^/CD8^+^ T‐cell ratios. It is important to note that measuring PBMC counts and assessing CD4^+^/CD8^+^ T‐cell ratios have not been determined to be the best measure of effectiveness of MPA in cats, but to date, limited information is available. Several factors could have contributed to the lack of T lymphocyte response to the PO MMF. One possibility is that MMF may not have been given at a high enough PO dosage. However, our results suggest that a dosage ≥15 mg/kg would not be well tolerated by cats. Additionally, MMF may not have been given for a long enough time period, particularly considering that MPA has a cytostatic effect.^5^, ^28^ Recent studies performed in healthy dogs suggest that MMF may need to be given for at least 2 weeks before decreased lymphocyte proliferation is observed.^31^ Interestingly, there is evidence that over time, a cumulative effect may occur after MMF administration, or initially other inhibitory actions on the immune system may occur, such as targeting key functions of dendritic cells.^15^, ^32^ In human medicine, conflicting evidence exists regarding the best pharmacodynamic marker after MMF administration, including, assessing disease activity scores, quantifying MPA plasma concentrations, and measuring inhibition of IMPDH activity or the concentrations of MPA in PBMC.^21^, ^33^ Currently, a validated method to measure IMPDH activity in cats is not available, and little research has been done to evaluate the effect of MPA on PBMC in cats. Previous studies have documented an effect of MMF on feline lymphocyte proliferation in vitro.^28^ Further studies assessing lymphocyte proliferation after PO dosing may help clarify MMF's effects in cats.

Another factor that could have contributed to the lack of effect on the lymphocyte counts is variability in the MPA target of IMPDH in certain cats. Recently, investigations in human medicine suggest that single nucleotide polymorphisms (SNPs) in genes encoding IMPDH may influence the inhibitory activity of MPA.^34^ Unfortunately, we did not assess the genetic background of our cats or whether or not genetic polymorphisms in genes encoding for IMPDH could have contributed to our findings. However, SNPs in genes encoding IMPDH should be considered in future studies.

In conclusion, we obtained novel information regarding the disposition of MPA and its effects on total PBMC and CD4^+^/CD8^+^ T‐cell ratios after PO MMF administration in healthy cats. Because of variability in tolerance to MMF and current information on its effect on lymphocytes in vitro, MMF cannot be recommended for the routine treatment of immune‐mediated disorders in cats.

## CONFLICT OF INTEREST DECLARATION

Authors declare no conflict of interest.

## OFF‐LABEL ANTIMICROBIAL DECLARATION

Authors declare no off‐label use of antimicrobials.

## INSTITUTIONAL ANIMAL CARE AND USE COMMITTEE (IACUC) OR OTHER APPROVAL DECLARATION

The research protocol was approved by the Washington State University IACUC (ASAF # 04665‐005).

## HUMAN ETHICS APPROVAL DECLARATION

Authors declare human ethics approval was not needed for this study.
